# Glutaminase is essential for the growth of triple-negative breast cancer cells with a deregulated glutamine metabolism pathway and its suppression synergizes with mTOR inhibition

**DOI:** 10.1371/journal.pone.0185092

**Published:** 2017-09-26

**Authors:** Michael Lampa, Heike Arlt, Timothy He, Beatriz Ospina, Jason Reeves, Bailin Zhang, Joshua Murtie, Gejing Deng, Claude Barberis, Dietmar Hoffmann, Hong Cheng, Jack Pollard, Christopher Winter, Victoria Richon, Carlos Garcia-Escheverria, Francisco Adrian, Dmitri Wiederschain, Lakshmi Srinivasan

**Affiliations:** 1 Oncology, Sanofi, Cambridge, MA, United States of America; 2 Integrated Drug Discovery Platform, Sanofi, Waltham, MA, United States of America; 3 Research Platforms, Sanofi, Vitry-Sur-Seine, Vitry, FRANCE; University of South Alabama, UNITED STATES

## Abstract

Tumor cells display fundamental changes in metabolism and nutrient uptake in order to utilize additional nutrient sources to meet their enhanced bioenergetic requirements. Glutamine (Gln) is one such nutrient that is rapidly taken up by tumor cells to fulfill this increased metabolic demand. A vital step in the catabolism of glutamine is its conversion to glutamate by the mitochondrial enzyme glutaminase (GLS). This study has identified GLS a potential therapeutic target in breast cancer, specifically in the basal subtype that exhibits a deregulated glutaminolysis pathway. Using inducible shRNA mediated gene knockdown, we discovered that loss of GLS function in triple-negative breast cancer (TNBC) cell lines with a deregulated glutaminolysis pathway led to profound tumor growth inhibition *in vitro* and *in vivo*. GLS knockdown had no effect on growth and metabolite levels in non-TNBC cell lines. We rescued the anti-tumor effect of GLS knockdown using shRNA resistant cDNAs encoding both GLS isoforms and by addition of an α-ketoglutarate (αKG) analog thus confirming the critical role of GLS in TNBC. Pharmacological inhibition of GLS with the small molecule inhibitor CB-839 reduced cell growth and led to a decrease in mammalian target of rapamycin (mTOR) activity and an increase in the stress response pathway driven by activating transcription factor 4 (ATF4). Finally, we found that GLS inhibition synergizes with mTOR inhibition, which introduces the possibility of a novel therapeutic strategy for TNBC. Our study revealed that GLS is essential for the survival of TNBC with a deregulated glutaminolysis pathway. The synergistic activity of GLS and mTOR inhibitors in TNBC cell lines suggests therapeutic potential of this combination for the treatment of vulnerable subpopulations of TNBC.

## Background

Altered energy metabolism is one of the recognized hallmarks of cancer [1]. Tumor cells harbor the ability to switch to alternate sources of energy in order to sustain their energy requirements in a nutrient-poor microenvironment [2, 3]. The amino acid glutamine is one source often used by cancer cells to support their rapid growth and proliferation [4, 5]. In tumors, glutamine is involved in a number of intracellular processes including ATP generation and the biosynthesis of proteins, lipids, and nucleic acids and acts as a key regulator of cellular redox status [6, 7]. The intracellular processing of glutamine begins with its catalysis to become glutamate (Glu) by the mitochondrial enzyme glutaminase (GLS). The subsequent deamination of Glu generates αKG, a tricarboxylic acid (TCA) cycle intermediate. In mammals, two genes, *GLS* and *GLS2*, encode distinct forms of glutaminases. The KGA and GAC isoforms generated by alternative splicing are derived from the *GLS* gene and share an identical N-terminus and catalytic domain but have distinct C-termini of unknown function [8, 9]. The GAC isoform is found to be overexpressed in tumors especially in breast cancer wherein the extent of its abundance correlates strongly with the tumor’s degree of malignancy [10–12]. Both KGA and GAC are phosphate (Pi) activated glutaminases. It has been postulated that Pi concentrations increase in the mitochondria under hypoxic conditions as experienced by many tumors thus prompting activation of GLS [6, 12].

Although glutamine has been shown to be an essential amino acid in rapidly dividing tumor cells, mutations or amplifications in the glutamine metabolism genes have not been identified. However, it has been found that genetic alterations in KRAS and MYC signaling pathways influence the expression and activity of GLS [13]. MYC exerts its effects through the microRNAs miR-23a and miR-23b that have binding sites in 3’UTR of GAC [14–16]. Cells transformed by mutant KRAS demonstrate increased expression of glutamine metabolism genes and become reliant on external sources of glutamine [17–19]. It has been reported that proliferation of KRAS mutant pancreatic ductal adenocarcinoma cells depend on glutamine metabolism, which is driven by GLS and downstream transaminases GOT1/2 [20, 21]. GLS has also been shown to be a direct effector of RHO-mediated transformation of breast cancer cells [10]. In addition, synthetic lethal interactions of glutamine metabolism have been reported. For instance, glioblastoma or acute myeloid leukemia (AML) tumors that harbor IDH1/ IDH2 mutations are particularly dependent on the function of the GAC isoform for the anaplerotic replenishment of αKG, which is the source material used to generate the onco-metabolite 2-HG by these mutant enzymes [22–25]. Furthermore, the glutamine transporter ASCT2 has been found to be vital for triple-negative, basal-like breast cancer cell growth [26].

A critical gateway enzyme in glutaminolysis, GLS has been a sought after therapeutic target for small molecule inhibitors. The earliest approaches were based on glutamine mimetic antimetabolites DON, acivicin, and azaserine [27–29]. Despite modest preclinical antitumor activity, severe toxicity issues led to discontinuation of the clinical development of these molecules [30]. In the last 12 years, two novel glutaminase inhibitors, BPTES and 968, have been profiled extensively in the literature. Both agents specifically inhibit the GLS isoenzyme (both splice variants KGA and GAC) by binding to the protein at distinct allosteric sites and have demonstrated antitumor activity in multiple tumor types [31–35]. Very recently, the structural analogs of BPTES, CB-839 and AGX-4769, were found to be more potent GLS inhibitors [36, 37]. CB-839 (Calithera Biosciences) is currently being evaluated in multiple Phase I clinical trials in solid and hematological malignancies as a single agent and in combination with an immune checkpoint inhibitor (NCT02071888, NCT02071862, NCT02071927 and NCT02771626).

In this study, we validated GLS as a therapeutic target in TNBC cells using GLS specific shRNA constructs. We demonstrated that inducible knockdown of GLS in glutamine dependent TNBC cell lines leads to a decrease in downstream metabolite levels and profound inhibition of cell growth. Metabolite modulation and subsequent anti-proliferative effects induced by GLS knockdown were rescued by both genetic tools and supplementation with αKG, a metabolite downstream of GLS. Our findings *in vitro* were recapitulated *in vivo* as inducible knockdown of GLS in tumor xenografts resulted in a similar change in metabolite levels, suppressed tumor growth or tumor regression. Furthermore, using CB-839 as a pharmacological tool, we demonstrated that inhibition of GLS leads to a decrease in mTOR activity and an increase in the ATF4 stress response pathway only in responder breast cancer cell lines, suggesting that these molecular changes may be utilized as predictive PD/efficacy biomarkers for GLS inhibitor treatment. Lastly, we demonstrated that simultaneous inhibition of GLS and mTOR is synergistic in responder lines signifying a novel combination approach for the development of GLS inhibitors in basal-type breast cancers.

## Methods

### Ethics approval and consent to participate

Institutional Animal Care and Use Committee (IACUC) at Sanofi approved all experimentations with mice in the study.

### Cell lines and reagents

MDA-MB-231, MDA-MB-453, and SKBR3 were obtained from the American Type Culture Collection (Baltimore MD, USA). The MDA lines were grown in Dulbecco's Modified Eagle Medium, 4.5mg/mL glucose with 4mM glutamine plus 10% heat-inactivated fetal bovine serum while SKBR3 was cultured in McCoy’s 5A media plus 10% heat-inactivated fetal bovine serum. All three cell lines were incubated in 37°C, 5% CO_2_ conditions. SUM-159PT was obtained from Asterand Biosciences (Detroit MI, USA) and maintained in Ham's F-12 Media containing 10mM HEPES, 1μg/mL hydrocortisone solution, 5μg/mL human insulin and 5% heat-inactivated fetal bovine serum in 37°C, 5% CO_2_ conditions. The lentivirally transduced versions of these lines were grown in the same media but supplemented with tetracycline-free heat-inactivated fetal bovine serum (Clontech) and 1μg/mL puromycin. Culture conditions for the above cell lines were maintained in knockdown/genetic rescue assays. CB-839 was synthesized as described [38].

### Generation of stable cell lines

To knockdown the expression of human GLS, two lentiviral GLS-shRNA constructs from Sigma-Aldrich were utilized: TRCN0000051135 and TRCN0000051136. These constructs target both KGA and GAC isoforms of GLS. Each shRNA was inserted into the shRNA expression vector, pLKOpuro_U6-TO_TetR. A scrambled Null shRNA sequence (5’ ACCGTACGTTACGCGTAATGTTTCAAGAGAACGTTACGCGTAACGTACGGT-3’) was inserted into the same expression vector to serve as a non-targeting control. These constructs were packaged in formulation with Opti-MEM, Fugene 6 (Promega, E2691), the expression plasmid pCMVΔR 8.91 (Life Technologies), and PLD VSV-G (Life Technologies). The packaged virus was multiplied in HEK-293T cells. The supernatants were then harvested and used to infect the candidate lines. GLS shRNA stable cell lines were selected in media containing puromycin (1μg/mL).

To rescue the knockdown phenotype, shRNA-resistant lines of both KGA and GAC isoforms were generated from the GLS shRNA stable cell lines. pDONR221 (Thermo Fisher Scientific, 12536017) vector was used to produce entry clones for both isoforms. Gateway technology (Thermo Fisher Scientific) was utilized to produce the constructs in pLenti 6.3 cV5_DEST and pLCMVhyg-wpre_DEST expression vectors for KGA and GAC, respectively. The CMV promoter in both constructs is responsible for driving constitutive expression of the target gene. For both KGA and GAC isoforms, fifteen silent mutations were introduced in the following sequence regions: C1212A, A1215T, C1218T, T1224C, T1227A, A1230C, T1231C, C1347T, T1350A, A1353G, A1356T, T1359C, C1360A, A1362G, and T1365C. The same packaging and infection methodology used to produce the GLS shRNA stable cell lines was employed to incorporate shRNA-resistant KGA and GAC cDNA. Blasticidin (10μg/mL) and hygromycin (500μg/mL) selection markers were added in the media to isolate these rescue lines.

To induce GLS knockdown, 2.0 x 10^6^ cells from each GLS shRNA stable cell line were passaged into two T150 cm^2^ flasks either in the presence or absence of 1mg/mL doxycycline in media containing tetracycline-free fetal bovine serum. After three days incubation, the cells were trypsinized, counted, and apportioned based on the needs of the investigational assays to be performed.

### Cell proliferation and rescue

A bulk volume of cells was diluted to accommodate a seeding density of 2,000 cells/100μL (MDA-MB-231, MDA-MB-453, SUM-159PT) and 3,000 cells/100μL (SKBR3) in four 96-well white, clear bottom cell culture plates. To measure proliferation, each plate was incubated in the presence or absence of doxycycline and assayed with CellTiter-GLO (Promega) at 0, 3, 6, and 9 days after 3 days of doxycycline induction. On the day of analysis, a white plate backing was adhered to the bottom of each plate following microscopic examination. 100μL CellTiter-GLO reagent was added to each well and read in the Envision Multilabel Reader (Perkin Elmer). The unused cells were pelleted and frozen in -80°C for western analysis (1x10^6^ cells) and qRT-PCR (100,000 cells) to confirm knockdown. The metabolic rescue of GLS knockdown required adaptation of the cell proliferation assay procedure by adding 4mM Dimethyl 2-Oxoglutarate to the growth media of doxycycline induced GLS shRNA stable cell lines.

### Apoptosis

Apoptosis in the doxycycline induced GLS shRNA stable cell line MDA-MB-231 was measured using the PE Annexin V Apoptosis Detection Kit I (BD Biosciences Cat. No. 559763). After 3 days of doxycycline induction, the cells were trypsinized, counted, and reseeded in 10cm^2^ plates at a density of 20,000 cells/mL and incubated in 37°C, 5% CO_2_ for an additional 3 days. Following incubation, the cells were harvested by trypsinization and treated according to the Apoptosis Detection Kit protocol. One day before the cells were assayed for apoptosis, 1μM staurosporine was added to select wells to serve as a positive control. The samples were run on the FACScalibur flow cytometer (BD Biosciences) using CellQuest software (BD Biosciences). 20,000 events were acquired from each sample for analysis.

### qRT-PCR

mRNA levels were measured using TaqMan® Gene Expression Cells-to-CT™ Kit (Life Technologies Cat. No. AM1729). The procedure adhered to the manufacturer’s protocol with few changes. The frozen pellets were lysed in 50μL volumes with DNase I. For reverse transcription, each lysate was diluted 1:5 in a 50μL volume to make the cDNA. 5μL of cDNA per sample was mixed in a 1:5 dilution (25μL total) for gene amplification by PCR. Relative expression levels were determined using the 2^ΔCt^ method.

### Mass spectrometry

Following a 3-day doxycycline induction of shRNA expression, the cells were trypsinized, counted, and seeded into 10cm^2^ dishes at 1 x 10^5^ cells/ml. After 24 hours, the cells were washed with 1X PBS and supplanted with glutamine-free media containing 10% heat-inactivated dialyzed fetal bovine serum with 4mM U^13^- C_5_ labeled L-glutamine or 4.5g/L i U^13^- C_6_ labeled glucose in the presence or absence of doxycycline. The cells were left to incubate overnight at 37°C, 5% CO_2_. On the day of harvesting, an aliquot of media was stored in -80°C.

To capture intracellular metabolites, the cells were washed twice with PBS and collected in methanol:water (80:20) solution. The samples were centrifuged and the supernatants containing intracellular metabolites were collected. The pellets were resuspended in ice cold methanol:water (80:20) solution and centrifuged again. The supernatants were collected and pooled with the previous supernatant collection. The combined solutions were dehydrated by speed vacuum and reconstituted in 500μL water.

For metabolite analysis, all samples were derivatized with AccQ Fluor Reagent Kit from Waters (WAT052880, Waters). The resulting solutions were further diluted 200-fold for LC/MS/MS analysis. LC/MS/MS analysis was done on AB Sciex Qtrap 5500 coupled with Agilent Infinity 1290 UHPLC system at ES positive mode and scheduled MRMs. Waters AcQuity HSS T3 column (2.1X100mm, 1.8μm) was used for the separation. LC Gradient was run from 95% Mobile A (0.1% acetic acid in water) to 95% Mobile B (0.1% acetic acid in 1:1 MeOH/CAN) in 10 minutes. All derivatized amino acids were detected through monitoring the product ion of 171.1 m/z.

### Immuno blot analysis

The cells were collected, washed in PBS, and pelleted before lysing with RIPA buffer (Cell Signaling Technologies) containing protease and phosphatase inhibitors. After thorough homogenization, the protein lysates were generated following centrifugation (12,000rpm x 10min) and quantified using the DC Protein Assay (Bio-Rad Laboratories). Western blotting analysis was performed according to standard procedures. Antibodies used in western analyses: isoform specific antibodies for KGA (H00002744-A01, Novus Biologicals LLC) and GAC (19958-1-AP, Proteintech Group Inc.); antibodies specific for GAPDH (2118, CST), ATF4 (11815, CST), phospho-4E-BP1 (9455, CST), phospho-p70 S6Kinase (9234, CST), and phospho-S6 (2211, CST); and goat anti-mouse immunoglobulin G (115-036-003, Jackson ImmunoResearch Laboratories), goat anti-rabbit immunoglobulin G (115-036-003, Jackson ImmunoResearch Laboratories), and HRP-conjugated goat anti-mouse immunoglobulin G (115-036-003, Jackson ImmunoResearch Laboratories) secondary antibodies.

### In vivo xenografts

Institutional Animal Care and Use Committee (IACUC) at Sanofi approved all experimentations with mice in the study.

5 x 10^6^ cells/mouse were suspended in 0.2mL of 50% matrigel, 50% DPBS and injected subcutaneously into the flanks of female athymic nude mice (Harlan Laboratories). Mice were monitored for appearance of tumors. Tumor size was measured by caliper. Once the tumor volumes reached approximately 200mm^3^, mice were randomized and shifted to a diet containing doxycycline (400ppm). The entire study was terminated when the tumor volume in the control group reached 1200mm^3^. Up to 10 mice were in each group. Tumor samples were harvested from all mice for metabolite, qRT-PCR, and western blot analysis.

### TCGA analysis

Expression data for The Cancer Genome Atlas (TCGA) breast cancer samples was analyzed in Nextbio Clinical and exported for visualization in Spotfire. Relative expression of *GLS* and *GLUL* was calculated from RNA-Seq RPKM values as the fold change around the mean of all breast cancer samples using a z-score transformation of the expression of each gene. Classification within subtypes was provided by Nextbio and based on the PAM50 classifier [39].

### CB-839 compound treatment assays

Cells were seeded at a density of 1 x 10^6^ cells in 10mL Leibovitz (L-15) media (Life-Technologies) with 10% heat-inactivated FBS in 10cm^2^ culture dishes and incubated in 37°C in the absence of CO_2_. After 24 hours, CB-839 compound was added and incubated for an additional 72 hours in 37°C in the absence of CO_2_. After 72 hours post-treatment, the cells were washed once in PBS, placed on ice, scraped in 1mL ice-cold PBS, and aliquoted in micro-centrifuge tubes. The tubes were spun at 3,000rpm for 2 minutes and supernatant aspirated before being stored in -80°C.

### Determination of synergy between compounds

The determination of synergy between compounds was performed according to the Ray Design methodology [40]. A matrix of concentrations was prepared to understand the interaction of two compounds at several fixed proportions in the mixture. Each proportion or constant ratio of concentrations between the two compounds corresponds to a “ray.” The ray design includes one ray for each single agent and five combination rays. For each combination ray, the effective fraction “f” is estimated. The effective fraction corresponds to the proportion defined in terms of unit of effect for each compound alone according to their respective IC50 values. Three experiments were performed for each cell line. For each experiment, three 384-well plates were utilized to allow use of triplicates for combination rays and sextuplicates for single agent rays.

### Statistical analysis

The combination index, Ki, based on the Loewe additivity equation was determined for each ray. The estimation of each Ki and its 95% confidence interval was done using a global non-linear model fitted with the NLMIXED procedure of SAS V9.2 software. Additivity was concluded when the confidence interval of the combination index (Ki) included 1. Significant synergy was concluded when the upper bound of the confidence interval of Ki was less than 1. Significant antagonism was concluded when the lower bound of the confidence interval of Ki was greater than 1.

## Results

### Glutamine pathway is deregulated in basal type breast cancer cells

**Fig 1:**
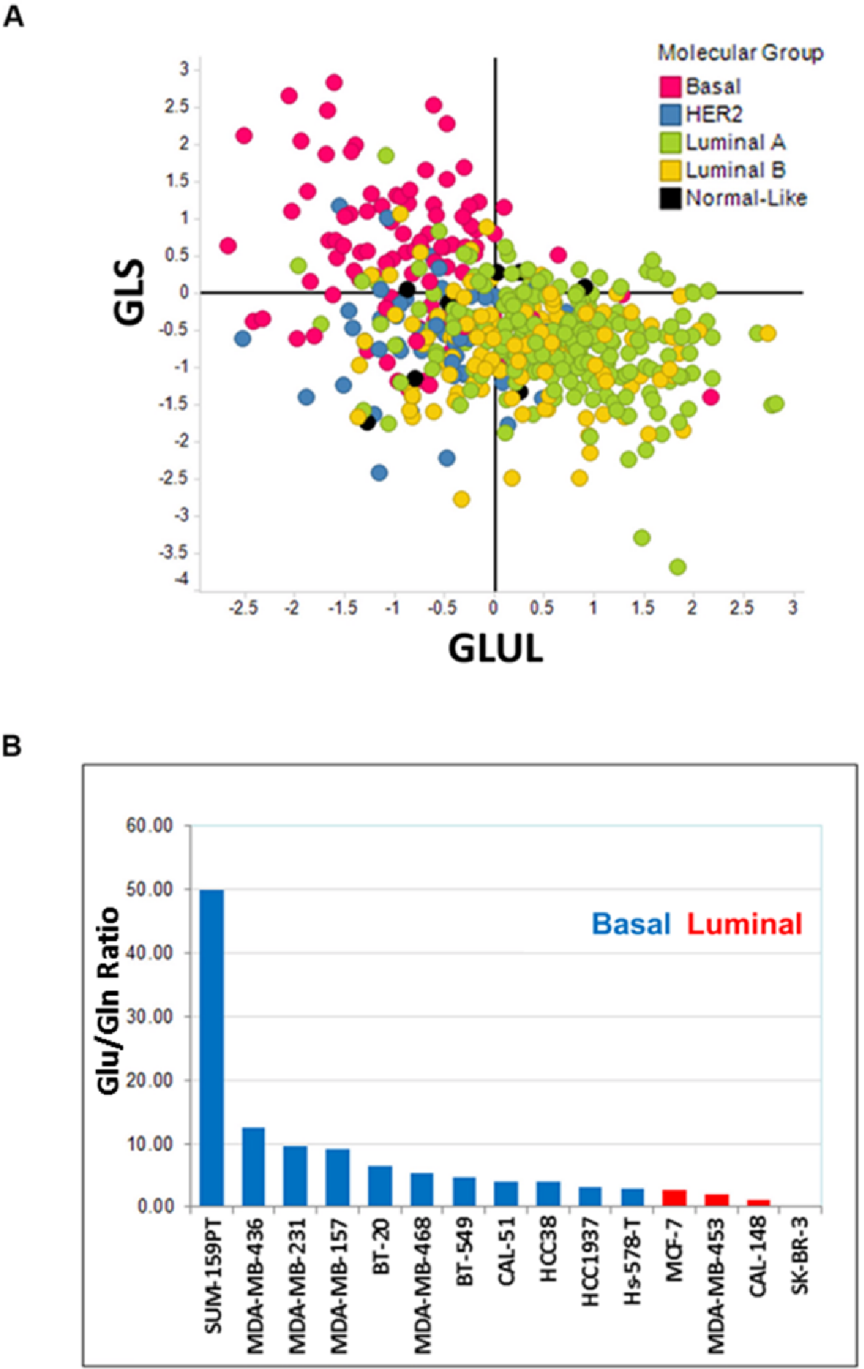
Basal breast cancers enrich for deregulation of glutamine metabolism pathway. Panel A. Scatter plot describing the relative gene expression levels of *GLS* and *GLUL* among breast cancers in the TCGA database. Data are plotted as log_2_ fold change around the mean of all breast cancer samples. Panel B. A total of 15 breast cancer cell lines were cultured in ATCC recommended media. Intracellular glutamine and glutamate levels were determined by mass spectrometry analysis. Data are plotted as a ratio of glutamate to glutamine levels and sorted from the highest to lowest values.

The ratio of glutamate to glutamine levels in cells is primarily controlled by two enzymes, glutaminase (GLS) and its anabolic counterpart glutamine synthetase (GLUL). To correlate glutamine levels with breast cancer subtype, we first analyzed the expression of *GLS* and *GLUL* as reported in The Cancer Genome Atlas (TCGA) database. Our analysis revealed that, unlike HER2 positive, Luminal A, Luminal B, or normal-like cancer types, many of the basal subtype breast cancer samples consistently displayed simultaneous up- and down- regulation of GLS and GLUL gene expression, respectively (Fig 1A). These findings indicated a deregulation of the glutaminolysis pathway in basal-like tumors confirming previous reports that ER and HER2 negative breast tumors possess the highest glutamate to glutamine (Glu/Gln) ratios [41, 42]. To further support the correlation between deregulated glutamine metabolism with breast cancer subtype, we measured Glu/Gln ratios in a panel of breast cancer cell lines from both basal and luminal breast cancer subtypes [43]. Consistent with the analysis derived from primary patient sample data, cell lines representing the basal subtype had increased Glu/Gln ratio when compared to cell lines representing the luminal subtype (Fig 1B). Based on these results, we selected cell lines representing both the basal and luminal subtypes to better understand the requirements of GLS for their growth and proliferation.

### GLS is essential for proliferation of TNBC cell lines *in vitro*

**Fig 2:**
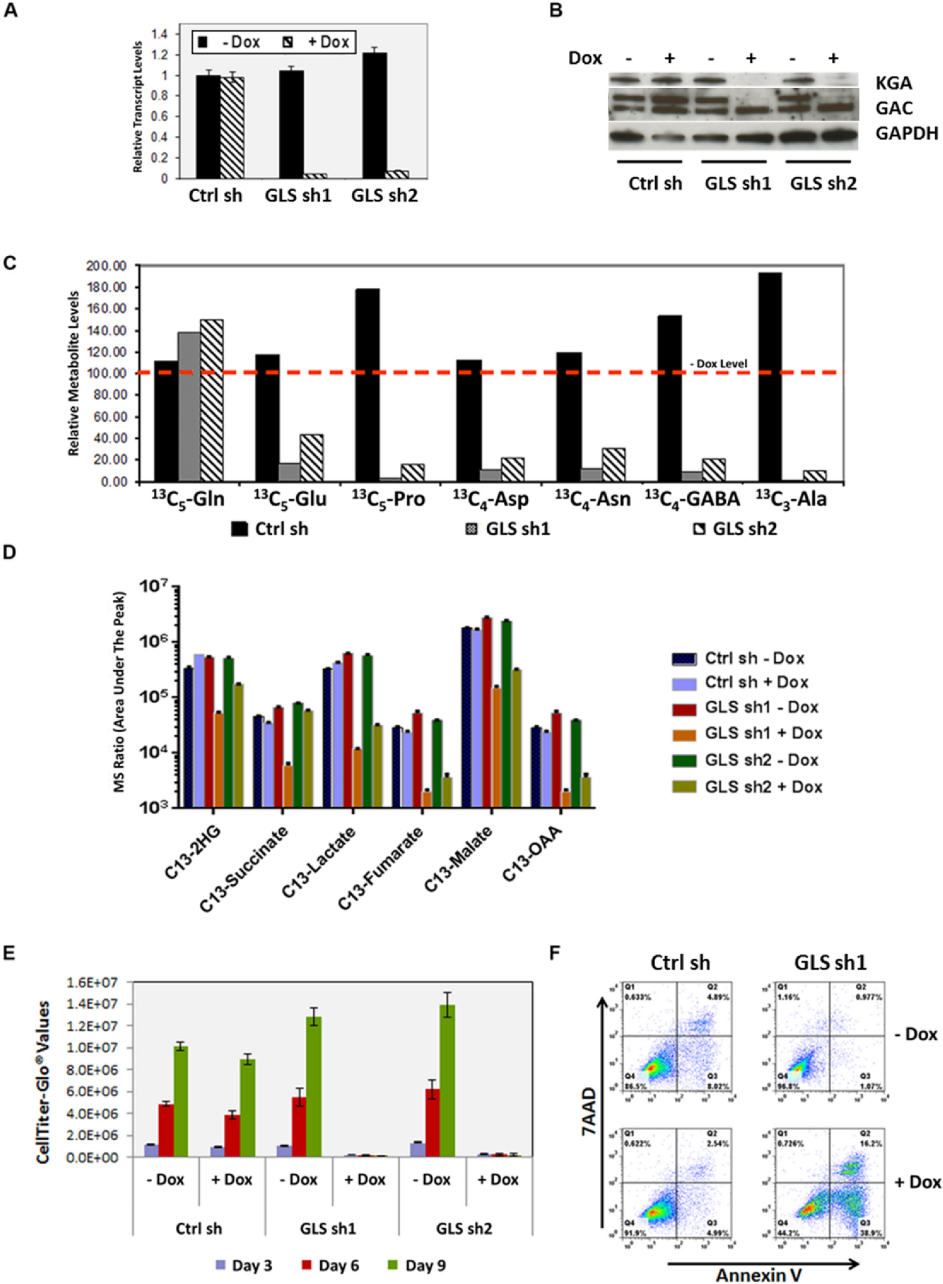
MDA-MB-231 breast cancer cell line requires GLS for growth and survival *in vitro*. Two inducible forms of GLS shRNAs, sh1 and sh2 were introduced into MDA-MB-231 cells. GLS knockdown was induced upon doxycycline addition. Panel A. Knockdown of *GLS* transcripts as measured by qRT-PCR. Panel B. Depiction of GLS protein knockdown by immunoblot analysis (Ab used-H00002744-A01, Novus Biologicals LLC and 19958-1-AP, Proteintech Group Inc.) at 72 hours post-doxycycline induction. Panel C. Incorporation of carbons from U^13^- C_5_ labeled L-glutamine into other non-essential amino acids as measured by mass spectrometry analysis after 24 hours of doxycycline induction. Data are normalized to a no doxycycline level and represented with a red dotted line indicating 100%. Panel D. Mass spectrometry analysis of TCA cycle intermediates upon GLS knockdown in MDA-MB-231 cells. Panel E. Relative proliferation of MDA-MB-231 cells upon GLS knockdown. Proliferation was measured using the CellTiter-GLO assay at the indicated time points post doxycycline induction. CellTiter-GLO values are plotted on the y-axis. The histogram plots describe the average and standard deviation of triplicate samples. Panel F. GLS knockdown induces apoptosis in MDA-MB-231 cells. 72 hours after doxycycline induction, cells were stained with Annexin V and 7-AAD and analyzed by flow cytometry for quantitation of apoptosis as illustrated by the FACS plots.

**Fig 3:**
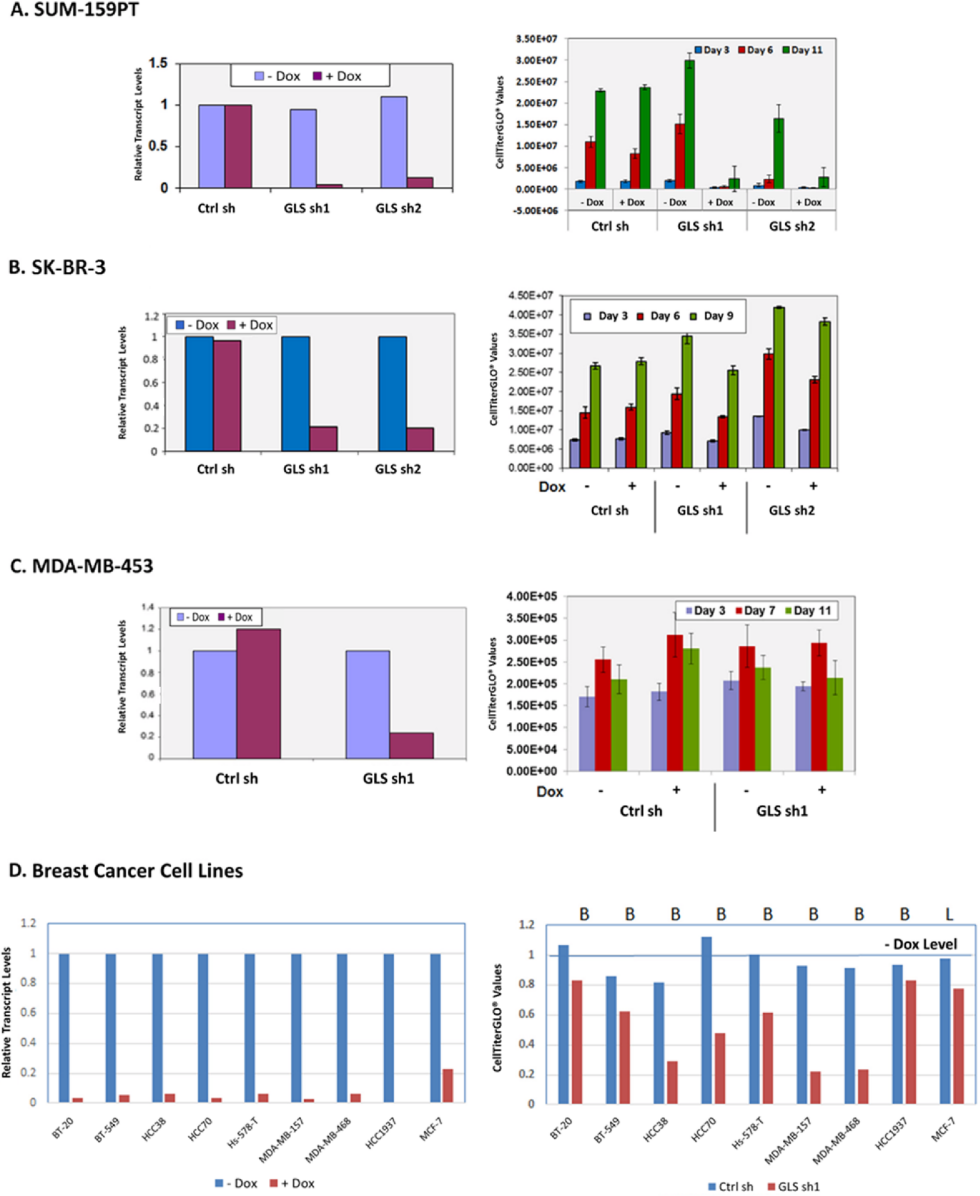
Assessment of GLS requirement for growth in multiple breast cancer cells. Two inducible GLS specific shRNAs, sh1 and sh2 were introduced in SUM-159PT (Panel A), SKBR3 (Panel B), MDA-MB-453 (Panel C) and 9 additional breast cancer lines (Panel D). Knockdown of *GLS* upon doxycycline induction was confirmed by qRT-PCR analysis (left). Cell proliferation as function of ATP generation was measured using the CellTiter-GLO assay (right).

To assess the functional requirement of GLS among various forms of breast cancer, we introduced doxycycline inducible shRNAs targeting GLS (sh1 and sh2) into cell lines representing TNBC and ER-positive breast cancers. Fig 2 shows the effects of doxycycline induced GLS knockdown in the TNBC cell line MDA-MB-231. GLS knockdown led to a profound decrease in GLS mRNA (panel A) and protein levels (panel B) which was accompanied by a significant reduction in the incorporation of glutamine derived carbon atoms into other non-essential amino acids (panel C) and TCA cycle metabolites (panel D). Cell proliferation was also markedly inhibited (panel E). In addition, the cells underwent significant apoptosis (panel F). Similar results were obtained with the TNBC cell line SUM-159PT (Fig 3A). In contrast, breast cancer cell lines of the luminal subtype, SKBR3 and MDA-MB-453, were not dependent on GLS for growth and proliferation (Fig 3B and 3C). We expanded this analysis to additional breast cancer cell lines (8 basal and 1 luminal) infected with the doxycycline inducible GLS shRNA construct sh1. In this panel, we identified four basal breast cancer lines—HCC38, HCC70, MDA-MB-468 and MDA-MB-157—that significantly depend on GLS for growth (greater than 50% decrease in proliferation). Two of the basal lines, BT-549 and Hs 578T, exhibited moderate dependency (40% decrease in proliferation). Two basal cell lines, BT-20 and HCC1937, along with the luminal cell line MCF7 were found to be insensitive to GLS knockdown (Fig 3D).

**Fig 4:**
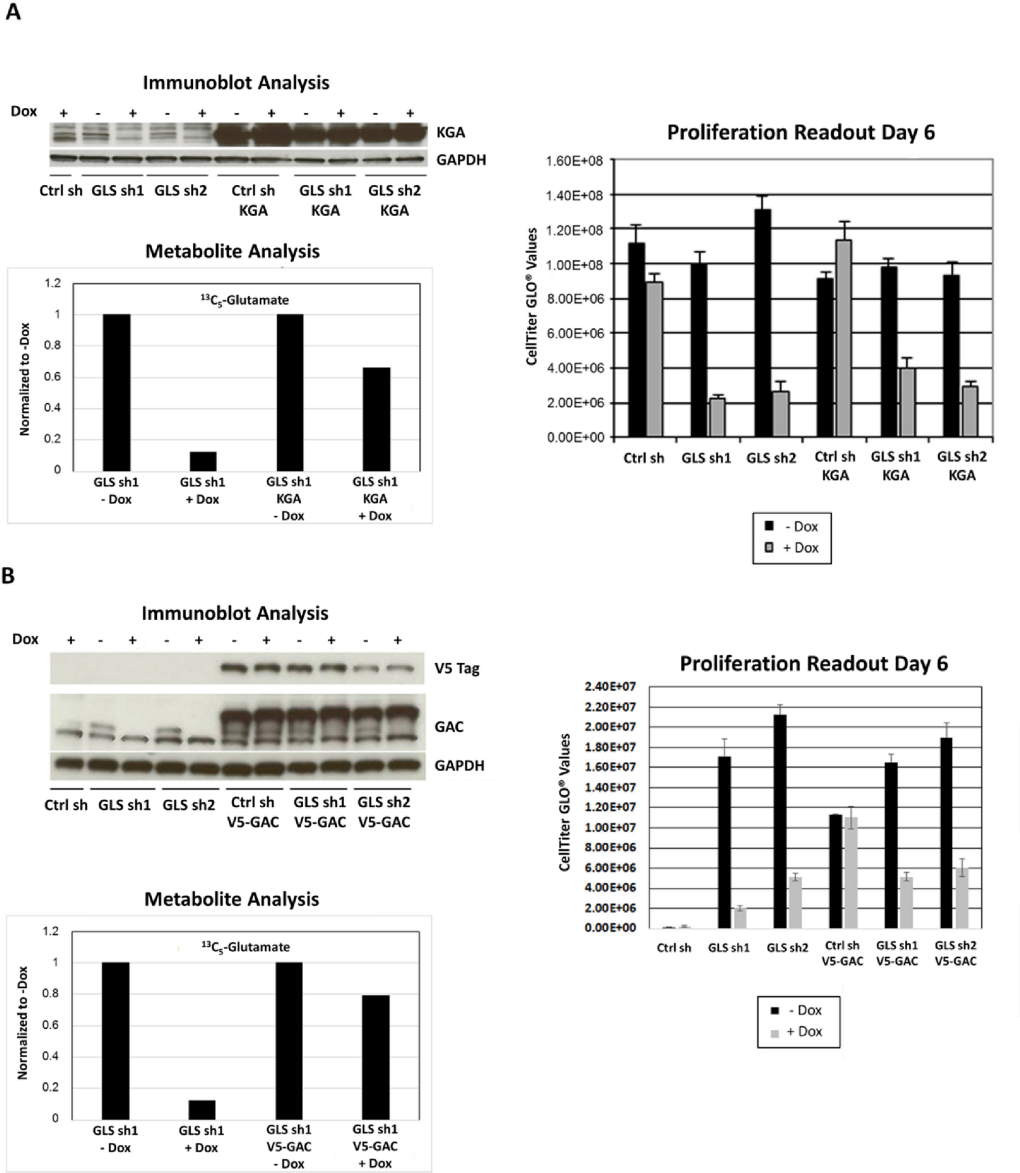
Single isoforms do not rescue GLS knockdown phenotype. Expression of either shRNA resistant KGA (Panel A) or GAC (Panel B) isoforms in MDA-MB-231. Doxycycline induction of GLS shRNA 1 and 2 effectively reduced endogenous levels of KGA and GAC isoforms. However, attempts to rescue the knockdown phenotype were unsuccessful when only an individual shRNA resistant isoform was introduced. Immunoblot analysis was performed 72 hours post doxycycline induction. Ctrlsh,GLS sh1, and GLS sh2 refer to cell lines with stably integrated Null shRNA or GLS shRNAs TRCN0000051135 and TRCN0000051136, respectively. Protein expression of shRNA resistant clones by immunoblot analysis using isoform specific antibodies, metabolite analysis by mass spectrometry and relative proliferation as measured by the CellTiter-GLO assay are displayed.

To validate the on-target activity of the shRNA against GLS, we designed phenotypic rescue experiments using both genetic tools and a cell permeable analog of a downstream metabolite of GLS, αKG. The GLS specific shRNAs, sh1 and sh2, used in our study targeted both the KGA and GAC isoforms, which was confirmed in MDA-MB-231 cell line (Fig 4). Immunoblot analysis revealed that the antibodies specific for KGA and GAC isoforms both recognize a doublet band around the expected molecular weight in MDA-MB-231 cell lysates. However, the levels of the faster migrating band of the doublet were not reduced upon doxycycline induction. This suggests the lower band of the doublets to be nonspecific and an inaccurate indicator of GLS protein expression upon GLS knockdown.

**Fig 5:**
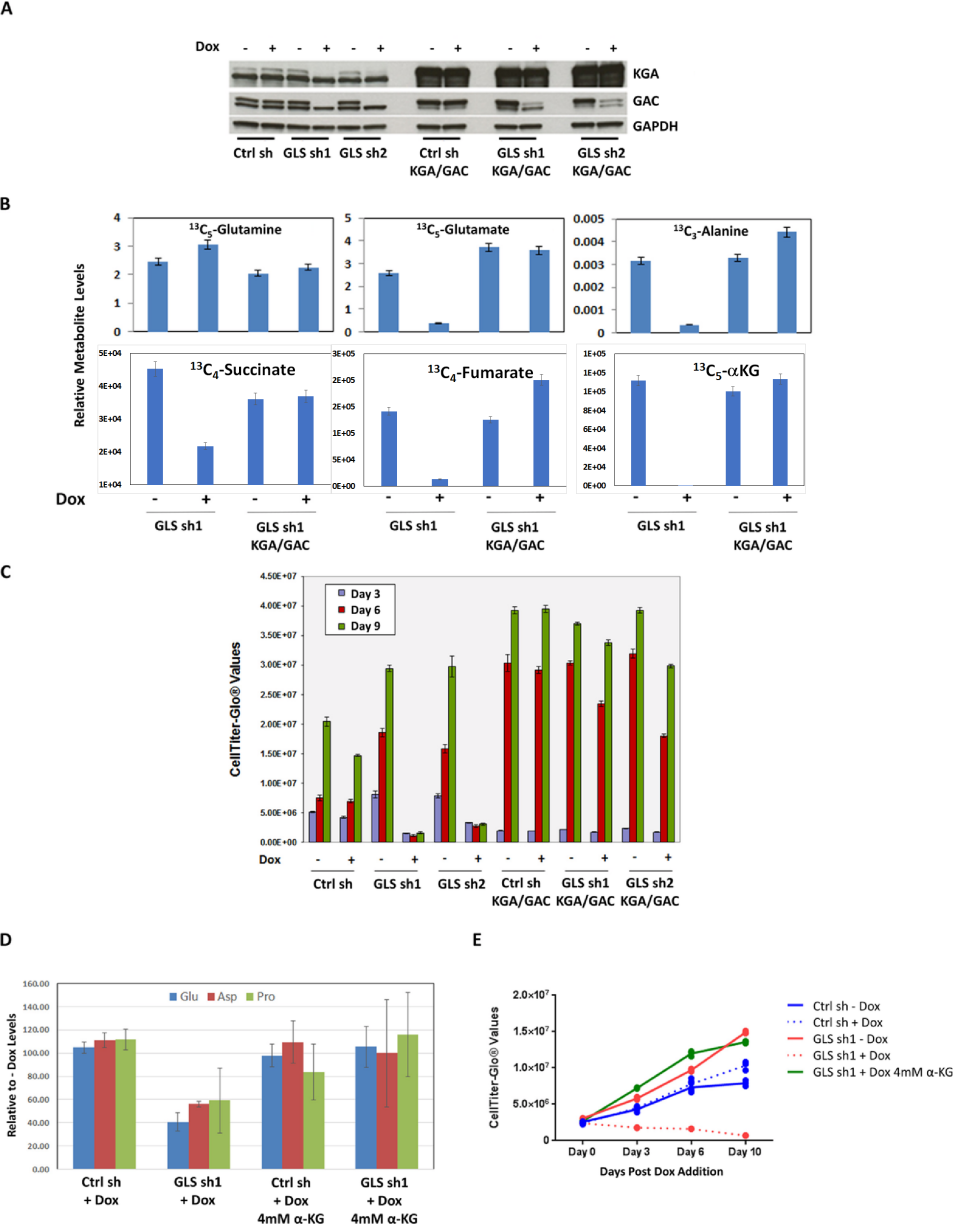
Genetic and metabolic rescue of GLS knockdown phenotype. Panel A: Expression of both shRNA resistant KGA and GAC isoforms in MDA-MB-231. Doxycycline induction of GLS shRNAs, sh1 and sh2 effectively reduced endogenous levels of KGA and GAC and were successfully rescued when both shRNA resistant isoforms were introduced. Immunoblot analysis was performed 72 hours post doxycycline induction. Ctrl sh, GLS sh1 and GLS sh2 refer to cell lines with stably integrated Null ShRNA or GLS shRNAs TRCN0000051135 and TRCN0000051136 respectively. Ctrl sh KGA/GAC, GLS sh1 KGA/GAC and GLS sh2 KGA/GAC also contain the stable integration of shRNA resistant versions of both KGA and GAC isoforms. Panel B: Expression of both shRNA resistant isoforms allowed for complete incorporation of U^13^- C_5_ labeled carbons from glutamine to downstream amino acids glutamate and alanine as well as TCA intermediates. Relative levels of the metabolites are plotted. Panel C. Expression of both shRNA resistant versions of KGA and GAC rescued the growth inhibition phenotype of the shRNAs. Proliferation was measured using the CellTiter-GLO assay at the indicated time points. The histogram plots described the average and standard deviation of triplicate samples. Supplementation with 4mM dimethyl αKG rescued the lack of incorporation of U^13^- C_5_ labeled carbons from glutamine in doxycycline induced shRNA stable cell lines (Panel D). The rescue of cell proliferation by αKG upon GLS shRNA knockdown was assessed using the CellTiter-GLO assay (Panel E).

Introduction of either shRNA resistant isoform led to almost 80% recovery of the downstream metabolite glutamate. However we were unable to achieve a full phenotypic rescue (Fig 4A and 4B), which suggested that both isoforms may be required to rescue GLS function. To successfully generate expression of both KGA and GAC isoforms simultaneously, we utilized expression vectors with different antibiotic selection markers. Inclusion of shRNA resistant cDNAs of both isoforms was necessary to completely rescue cell growth and reduced levels of glutamine derived metabolites upon GLS knockdown (Fig 5A, 5B and 5C). Complete rescue was also achieved by supplementation with a cell permeable analog of the downstream metabolite αKG (Fig 5 panels D and E). The results of these phenotypic rescue experiments firmly established the on-target specificity of the GLS shRNA and demonstrated that the effects of GLS knockdown reflect dependency on this target in basal breast cancer cell lines.

**Fig 6:**
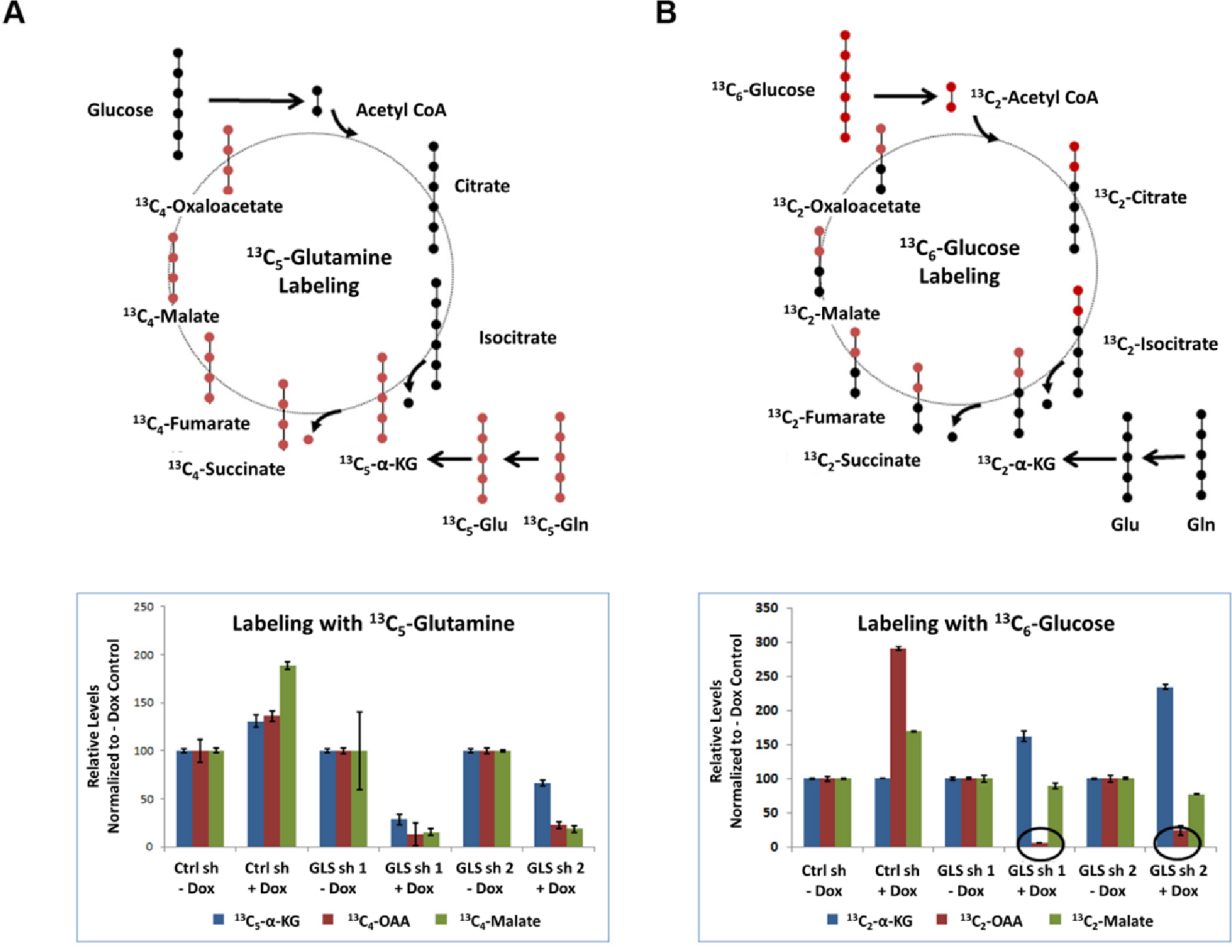
GLS is essential for intermediary metabolism in MDA-MB-231. Diagram representation of labelled ^13^C (red dots) and unlabeled ^12^C (black dots) from glutamine (Panel A) and glucose (Panel B) through the first TCA cycle is shown. Incorporation of the labelled carbon into TCA cycle intermediates upon GLS knockdown are shown in the bottom panels. Data acquired in triplicate are normalized to no doxycycline levels. Decrease in ^13^C_2_ labelled OAA seen with glucose labelling and GLS knockdown are indicated by the circles drawn.

To investigate the contribution of glutamine towards intermediary metabolism, we incubated GLS dependent cells MDA-MB-231 and SUM-159PT with either [U^13^- C_6_] glucose or [U^13^- C_5_] glutamine and determined the incorporation of each tracer into the TCA cycle upon GLS knockdown. A schematic of the tracer carbons through the first round of the TCA cycle is illustrated in Fig 6. Incorporation of glutamine derived carbons into TCA cycle metabolites αKG, malate, and oxaloacetate (OAA) was significantly reduced upon GLS knockdown in MDA-MB-231 cells (Fig 6A). However, when glucose was used as the source of labelled carbons, we observed a profound reduction in the levels of labelled OAA without a reduction of its preceding metabolite, malate, upon GLS knockdown (Fig 6B). These results suggest that breast cancer cells utilize OAA as a nutritive building block to generate other amino acids like aspartate through transaminase reactions in the absence of GLS. Profound reduction in aspartate levels upon GLS knockdown (Fig 2C) forced the cells to constantly process OAA to make up for the loss of aspartate thereby channeling this key intermediate away from the TCA cycle.

### GLS is required for the growth of TNBC xenografts

**Fig 7:**
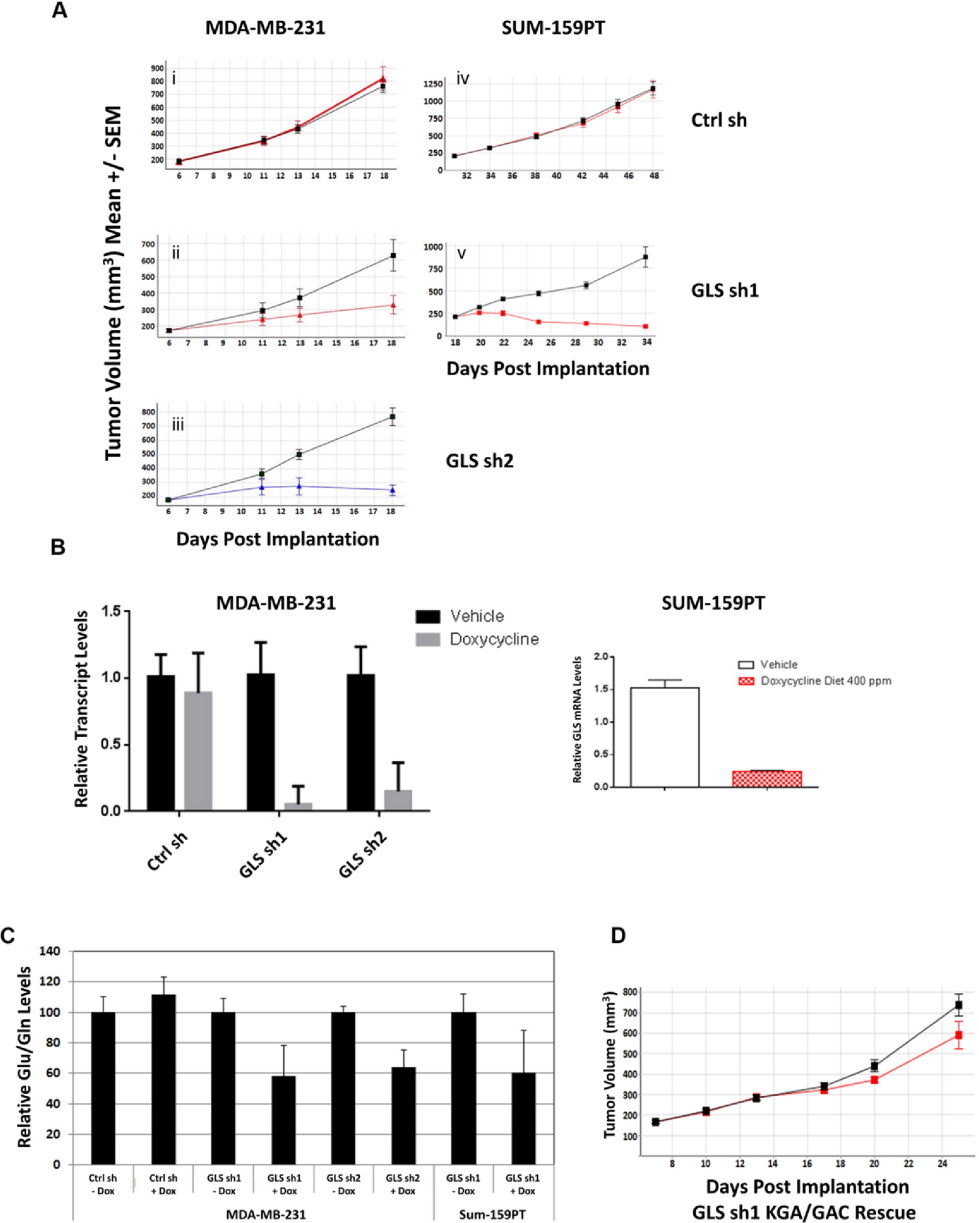
GLS is essential for the growth of TNBC tumor xenografts. Panels A (i-v) and D represent the growth curves of tumor xenografts for the doxycycline induced GLS shRNA stable MDA-MB-231 (i-iii) and Sum-159PT (iv and v) cell lines. The black line represents tumor growth in mice fed on a diet without doxycycline while the colored line represents tumor growth in mice fed on a diet with doxycycline. Panel B: qRTPCR analysis of *GLS* gene expression in MDA-MB-231 (left) and SUM-159PT GLS shRNA stable xenografts (right) confirming knockdown upon doxycycline induction. Panel C: Mass spectrometry analysis of glutamate to glutamine levels in MDA-MB-231 and SUM-159PT tumors. Data are normalized to no doxycycline levels. The histogram plots show the average and standard deviation of caliper measurements from 5 mice in each group. Panel D: Tumor growth depicting genetic rescue of GLS knockdown in doxycycline induced MDA- MB-231 GLS shRNA stable xenograft.

Once we established the requirement of GLS for the growth of TNBC cells *in vitro*, we tested whether GLS was essential for *in vivo* growth of these cell lines as tumor xenografts. Consistent with our *in vitro* results, doxycycline induced GLS knockdown led to profound tumor growth inhibition *in vivo*. GLS knockdown with sh1 and sh2 significantly reduced growth of MDA-MB-231 tumors and led to regression of SUM-159PT tumors (Fig 7A panels i-v). Intratumoral knockdown of GLS was confirmed by qRT-PCR analysis (Fig 7B) five days after introduction to a doxycycline diet. The ratio of glutamate to glutamine was also reduced in the tumors (Fig 7C) in line with our observations of GLS knockdown *in vitro*. Similar to the phenotypic rescue of GLS knockdown *in vitro*, incorporation of shRNA resistant cDNAs encoding for both GLS isoforms, KGA and GAC, resulted in a complete rescue of growth inhibition *in vivo*. (Fig 7D). These results clearly indicate that GLS is essential for the growth of these basal type breast tumors.

### GLS inhibition leads to activation of the integrated stress response (ISR) pathway

**Fig 8:**
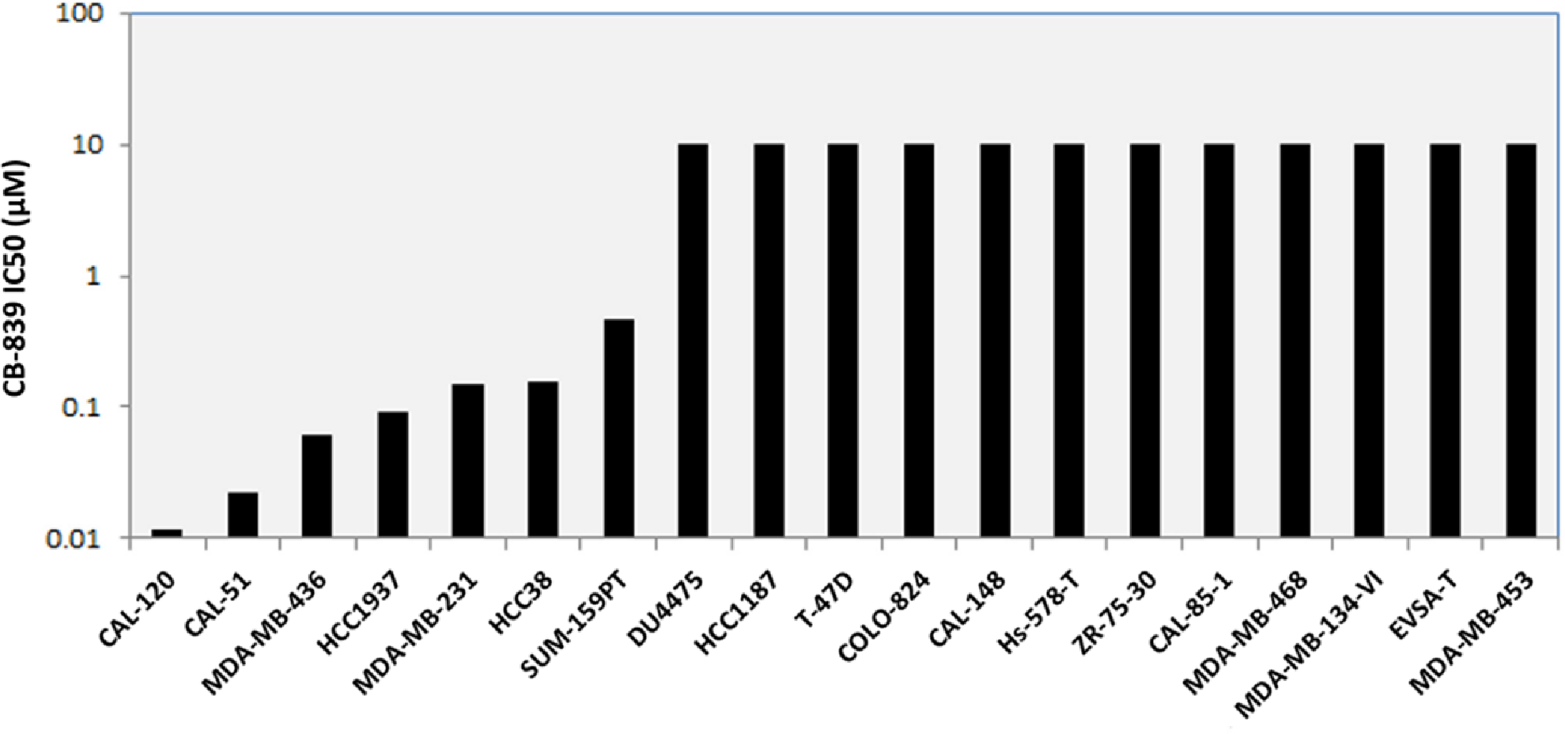
Growth inhibition activity of CB-839 across a panel of breast cancer cell lines. Cells were cultured in ATCC recommended medium and treated with varying concentrations of CB-839 to generate a 10- point dose response curve. Cell growth was measured after 72 hours incubation with CB-839 using the CellTiter-GLO assay. Maximum concentration of CB-839 used was 10μM. Cell lines refractory to compound treatment are plotted with an IC50 of 10μM.

**Fig 9:**
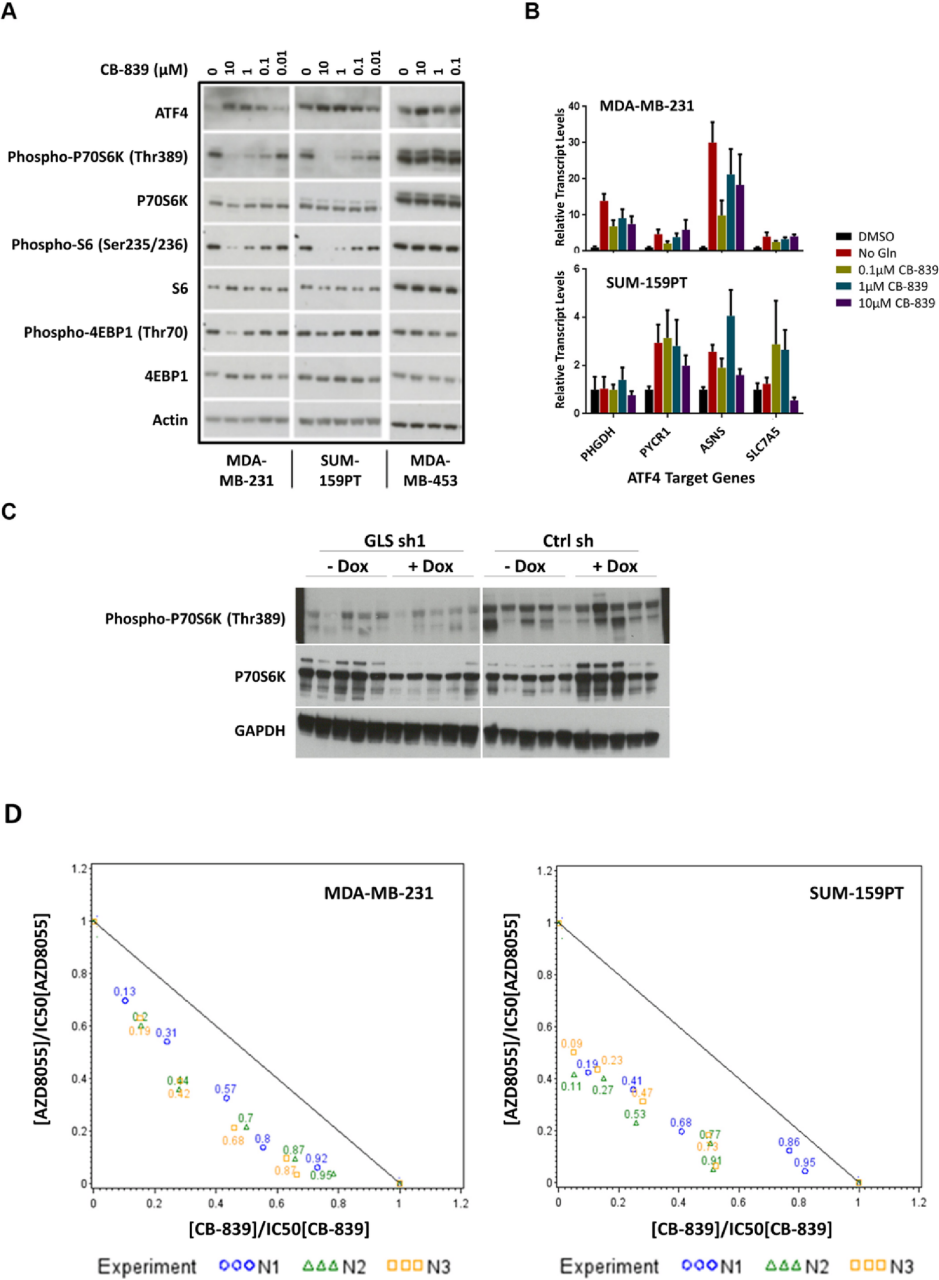
GLS inhibition activates the ISR pathway, inhibits the mTOR pathway, and synergizes with the mTOR inhibitor AZD8055. Panel A. Responders MDA-MB-231 and SUM-159PT and non-responder MDA-MB-453 cell lines were treated with increasing concentrations of CB-839 for 24 hours. Cell lysates were analyzed by immunoblot analysis with antibodies as indicated. Panel B. qRT-PCR analysis of MDA-MB-231 (top) and SUM-159PT (bottom) cells treated with varying concentrations of CB-839 or in the absence of glutamine in culture medium. Targets of ATF4 transcription factor as described were analyzed 24 hours after compound treatment. Data was acquired in triplicate and normalized to DMSO treated cells. Panel C. SUM-159PT xenograft lysates were analyzed by immunoblot analysis with antibodies as indicated. Each lane corresponds to xenografts from individual mice, 5 mice per treatment group. Panel D. Isobologram representing combined treatment of the GLS inhibitor CB-839 and mTOR inhibitor AZD8055 in MDA-MB-231 and SUM-159PT. The reference line joining the point (0,1) on the y-axis to the point (1,0) on the x-axis is a marker for additivity. The Ki values calculated from the combination rays in the experiment are found below the additivity line indicating synergy between the two compounds. Three Ki measurements corresponding to three independent experiments are plotted individually for each ray. The numbers in the plot represent the effective fraction of CB-839 in each combination ray.

Results of GLS knockdown experiments confirmed the pivotal role GLS plays in the proliferation of TNBC cell lines both *in vitro* and *in vivo*. To further validate these findings, we synthesized a recently characterized GLS inhibitor, CB-839 [36] and verified its activity in a panel of TNBC cell lines (Fig 8). We posited that responder cell lines inhibited by a GLS targeting compound should respond similarly to cells undergoing the stresses of nutrient starvation and therefore may result in the activation of the ISR pathway. ATF4 is the most well characterized transcription factor in this pathway and is preferentially translated upon phosphorylation of eIF2α. ATF4 is required for the optimal induction of other transcription factors ATF3 and ATF5 involved in the ISR pathway [44, 45]. In addition, MYC-mediated cell death that occurs upon glutamine deprivation of neuroblastoma cells has been shown to be facilitated by the ATF4 pathway [46]. Treatment of the GLS sensitive lines MDA-MB-231 and SUM-159PT with CB-839 led to accumulation of ATF4 protein in a dose dependent manner (Fig 9A). Upon induction, ATF4 regulates the expression of a large number of genes involved in amino acid synthesis and transport. Gene expression analysis of the responder lines MDA-MB-231 and SUM-159PT upon CB-839 treatment revealed induced expression of ATF4 targets PYCR1, ASNS and SLC7A5 (Fig 9B). SUM-159PT had a higher basal level of ATF4 protein and demonstrated a 2-3-fold increase in ATF4 target gene transcripts. The induction of ATF4 protein was more profound in the MDA-MB-231 cell line with a 5-30-fold increase in ATF4 target gene transcripts. Taken together, these findings demonstrate that GLS inhibition leads to the induction in ISR pathway activity in breast cancer cell lines.

### GLS inhibition leads to decrease of mTOR activity and synergizes with mTOR inhibitors

We also evaluated the effects of GLS inhibition on the mTOR pathway. The mTOR pathway serves as a central regulator of cellular metabolism and integrates both extracellular and intracellular signals. mTOR signaling is controlled by the availability of amino acids (reviewed in [47] and references therein). In the human sarcoma cell line U-2 OS, glutaminolysis has been shown to activate mTOR pathway by modulating GTP binding of RagB [48]. Upon treatment of breast cancer cell lines with GLS inhibitor CB-839, there was a decrease in the levels of phospho-P70 S6K (Thr 389) in the responder cell lines only (Fig 9A). We also observed a modest decrease in the levels of phospho-S6 and phospho-4EBP1 (Fig 9A). To correlate these findings with the GLS knockdown data in our xenograft model SUM-159PT, we analyzed these tumor lysates for both ATF4 levels and phospho-P70 S6K (Fig 9C). We detected a consistent decrease in the levels of phospho-P70 S6K five days after introduction to a doxycycline diet. We also noted a slight decrease in phospho-P70 S6K in tumors not exposed to doxycycline, suggesting knockdown of GLS due to low level expression of the shRNA in the absence of doxycycline.

Modulation of the mTOR pathway by the GLS inhibitor suggested the possibility that combination of CB-839 with mTOR inhibitors may result in a synergistic inhibition of cell growth. To determine potential synergistic effects, we tested two mTOR inhibitors, the allosteric mTOR inhibitor everolimus and the catalytic mTOR inhibitor AZD8055 [49], in combination with CB-839 in the TNBC cell lines MDA-MB-231 and SUM-159PT. As a single agent, everolimus did not inhibit *in vitro* growth nor did it enhance CB-839 activity. In contrast, the more potent mTOR inhibitor AZD8055 showed modest single agent activity and also demonstrated significant synergy with CB-839. Results of the combination experiments are summarized in Table 1 and the isobolograms are shown in Fig 9D.

**Table 1:** Combination index obtained for MDA-MB-231 and SUM-159PT treated with CB-839 and AZD8055. ‘F’ denotes effective fraction of CB-839 from the combination. ‘Ki’ denotes the calculated combination index. N1, N2, and N3 denote samples obtained from three independent experiments.

MDA_MB-231			SUM-159PT		
Experiment	f	Ki (confidence intervalat 95%)	Experiment	f	Ki (confidence intervalat 95%)
N1	0.09	0.7788 [0.6619; 0.9164]	N1	0.19	0.5229 [0.4650; 0.5880]
N2	0.10	0.8181 [0.6689; 1.0007]	N2	0.11	0.4679 [0.4168; 0.5253]
N3	0.11	0.6671 [0.5380; 0.8271]	N3	0.09	0.5520 [0.4947; 0.6161]
N1	0.23	0.8654 [0.7436; 1.0072]	N1	0.41	0.6068 [0.5319; 0.6922]
N2	0.25	0.7289 [0.6035; 0.8803]	N2	0.27	0.5523 [0.4893; 0.6235]
N3	0.26	0.5781 [0.4710; 0.7096]	N3	0.23	0.5661 [0.5107; 0.6275]
N1	0.47	0.6814 [0.5913; 0.7852]	N1	0.68	0.6062 [0.5166; 0.7113]
N2	0.49	0.6523 [0.5480; 0.7764]	N2	0.53	0.4872 [0.4186; 0.5670]
N3	0.51	0.5978 [0.4965; 0.7197]	N3	0.47	0.5930 [0.5241; 0.6711]
N1	0.73	0.6119 [0.5211; 0.7185]	N1	0.86	0.8913 [0.7416; 1.0712]
N2	0.75	0.6769 [0.5650; 0.8110]	N2	0.77	0.6567 [0.5495; 0.7849]
N3	0.76	0.5877 [0.4837; 0.7140]	N3	0.73	0.6829 [0.5952; 0.7835]
N1	0.89	0.7189 [0.6054; 0.8537]	N1	0.95	0.8643 [0.6988; 1.0690]
N2	0.90	0.7413 [0.6070; 0.9052]	N2	0.91	0.5654 [0.4612; 0.6930]
N3	0.90	0.6280 [0.5078; 0.7765]	N3	0.89	0.5870 [0.4951; 0.6960]

## Discussion

Recent progress towards understanding cancer metabolism has led to the identification of metabolic targets that could be of therapeutic interest. GLS is emerging as a potent target in the glutaminolysis pathway. Many cancer cells depend on glutamine as an energy source for growth and overall survival, yet mutations or amplifications that contribute to glutaminolysis pathway deregulation have not yet been identified or fully understood. This study finds that the *GLS* gene is necessary for the growth and survival of TNBC tumors *in vitro* and *in vivo*. We confirmed the significance of GLS in basal type breast cancer as evidenced by enhanced glutamine utilization and a higher GLS to GLUL ratio. In addition, metabolic analysis of cell lines representing the basal subtype corroborated our observations as all had a higher glutamate to glutamine ratio. Target validation in oncology is often limited by the number of suitable cell lines and parameters in which these lines are tested as they do not fully encompass the behavior of tumors in their natural microenvironment. In this study, we attempted to mitigate this problem by choosing cell lines that closely represent the human tumor with respect to target function.

The two major isoforms of GLS, KGA and GAC, are generated by alternate splicing at the C-termini with the KGA isoform being the longer of the two. Both variants have intact GLS enzyme domains and only KGA contains ankyrin repeats in its unique carboxy terminus. Both isoforms are reported to be mitochondrial and tissue specific [8]. The breast cancer cell lines used in our study express both isoforms. Furthermore, the shRNAs that we utilized targeted the catalytic domain of GLS therefore knocking down both isoforms. We discovered that neither the KGA nor GAC isoform alone was enough for complete phenotypic rescue. The requirement that both isoforms need be expressed simultaneously to elicit complete phenotypic rescue suggests that each isoform may either possess a distinct and essential function in the operability of GLS or both isoforms of GLS are required for providing adequate levels of glutaminase activity for high cell proliferation needs. For other tumor types, it has been reported that GAC is the predominant isoform that drives cancer cell growth [11, 50]. There is still much to be learned about these variants that requires further investigation. More importantly, we achieved phenotypic rescue upon supplementation with a cell permeable form of αKG thus confirming a reliance on glutamine as a major source of carbon to feed the TCA cycle in these breast cancer lines. The metabolite rescue also affirms that the anapleurotic generation of αKG through conversion from glutamate is the major glutamine catabolism pathway at play in these cells.

The small molecule GLS inhibitors and extensively validated GLS shRNA constructs used in our studies allowed us to demonstrate that pharmacological inhibition of GLS or its knockdown has a profound effect on the growth of breast cancer cells in both *in vitro* and *in vivo* settings, which has not been reported thus far [10, 36]. Our data implies that a sustained and complete inhibition of the enzyme is required to achieve marked therapeutic benefit targeting GLS,. Multiple GLS inhibitors described to date are broadly classified either as catalytic inhibitors that compete directly with glutamine or allosteric inhibitors that bind outside of the active site. Three allosteric inhibitors of GLS have been reported thus far—BPTES, compound 968, and CB-839. Treatment of GLS dependent breast cancer cells with compound 968 did not decrease the levels of TCA cycle intermediates nor inhibit growth under the conditions and concentrations tested. On the other hand, BPTES and its more potent analog CB-839 demonstrated the expected pharmacodynamic (PD) modulation, growth inhibition, and rescue phenotype by αKG in GLS dependent breast cancer cells. When profiled against a larger panel of breast cancer cell lines, CB-839 exhibited anti-proliferative activity against TNBC cell lines but not ER or HER2+ cell lines [36]. Consistent with our study, analysis of metabolite flux in primary breast cancer tissue revealed that TNBC tumors have a higher glutamate to glutamine ratio denoting increased GLS activity in TNBC cells [41, 51].

Based on our findings *in vitro*, we proceeded to test CB-839 for *in vivo* tumor growth inhibition on the same set of TNBC cell lines. We observed limited antitumor activity of the compound in MDA-MB-231 (ΔT/ΔC~63%) and SUM-159PT (ΔT/ΔC~69%) tumor xenografts. Although unexpected based on the phenotypes observed upon GLS knockdown with shRNA, the results do agree with the limited single agent anti-tumor activity of CB-839 reported in the literature [36]. This modest activity *in vivo* can perhaps be explained by the suboptimal pharmacokinetic properties of this compound in experimental models of cancer.

For cells that are highly dependent on glutamine as a major source of carbon and nitrogen for growth and survival, GLS knockdown or inhibition will likely imitate conditions of amino acid starvation and subsequently lead to activation of the ISR pathway and inhibition of the mTOR pathway. The mTOR pathway is a central regulator of cellular metabolism that promotes growth in response to nutrient availability [47]. The mTOR inhibitor AZD8055 has been shown to synergize with GLS inhibitor 968 in a glioblastoma model [52]. We observed synergy of CB-839 with the highly potent mTOR catalytic site inhibitor in breast cancer cell lines suggesting that therapeutic strategies for GLS inhibitors may include combinations with drugs targeting modulators in both pathways. The pathway interaction between glutamine catabolism and mTOR activity need to be further investigated in these basal breast cancer cell lines. Additional potential combination partners for GLS inhibitors could be targets within the ISR pathway. One example is GCN2, the inhibitor of eiF2αkinase that senses and responds to amino acid deficiency by binding to uncharged tRNAs [53]. Other potential combination partners for GLS inhibitors could include immune checkpoint inhibitors. Recent reports suggest increased antitumor efficacy in syngeneic mouse tumor models treated with both the GLS inhibitor CB-839 and either anti-PD1 or anti-PDL1 immune checkpoint inhibitors [54].

## Conclusion

Our work has clearly demonstrated that cell lines representing basal breast cancer subtype are highly sensitive to glutaminolysis inhibition that results in a profound decrease in TCA cycle intermediates and glutamine derived amino acids. Our analysis of the cellular responses to either GLS knockdown or pharmacological inhibition has identified potential PD markers and provides a molecular basis for combining GLS and mTOR inhibitors as a novel therapeutic strategy for treating basal breast cancers.

## Supporting information

S1 Checklist NC3Rs ARRIVE guidelines checklist filled.pdf. (PDF)

## Abbreviations

GLUL: Glutamine synthetase
GLS: Glutaminase 1
αKG: α-ketoglutarate
mTOR: mammalian target of rapamycin
ATF4: Activating transcription factor 4
TNBC: Triple-negative breast cancer
OAA: Oxaloacetate
ISR: Integrated stress response
